# The relationship between vitamin D level and second acid-fast bacilli (AFB) smear-positive during treatment for TB patients was inferred by Bayesian network

**DOI:** 10.1371/journal.pone.0267917

**Published:** 2022-05-04

**Authors:** Xiaoxu Zhang, Yan Zhang, Wenjun Xia, Yajie Liu, Hongkai Mao, Liangliang Bao, MingQin Cao

**Affiliations:** 1 Department of Epidemiology and Health Statistics, School of Public Health, Xinjiang Medical University, Urumqi, Xinjiang Uygur Autonomous Region, China; 2 Medical Record Room, Third Affiliated Hospital of Xinjiang Medical University, Urumqi, Xinjiang Uygur Autonomous Region, China; The University of Georgia, UNITED STATES

## Abstract

**Background:**

Vitamin D is related to human immunity, so we used Bayesian network model to analyze and infer the relationship between vitamin D level and the acid-fast bacilli (AFB) smear-positive after two months treatment among pulmonary tuberculosis (TB) patients.

**Methods:**

This is a cross-sectional study. 731 TB patients whose vitamin D level were detected and medical records were collected from December 2019 to December 2020 in XinJiang of China. Logistic regression was used to analyze the influencing factors of second AFB smear-positive. Bayesian network was used to further analyze the causal relationship among vitamin D level and the second AFB smear-positive.

**Results:**

Baseline AFB smear-positive (*OR* = 6.481, 95%*CI*: 1.604~26.184), combined cavity (*OR* = 3.204, *95%CI*: 1.586~6.472), full supervision (*OR* = 8.173, 95%CI:1.536~43.492) and full management (*OR* = 6.231, *95%CI*:1.031~37.636) were not only the risk factors and can also be considered as the reasons for second AFB smear-positive in TB patients (Ensemnle > 0.5). There was no causal relationship between vitamin D level and second AFB smear-positive (Ensemnle = 0.0709).

**Conclusions:**

The risk factors of second AFB smear-positive were baseline AFB smear-positive, combined cavity, full supervision and full management. The vitamin D level in TB patients was not considered as one of the reasons for the AFB smear-positive.

## 1 Background

Tuberculosis (TB) is still a major health problem in today’s world, especially in the developing countries of Asia, Africa and Latin America [1]. In the past twenty years, China has made significant progress in the prevention and treatment of tuberculosis. However, 866,000 people were still suffering from TB and 37,000 died of TB in 2018 [2]. The negative rate of sputum bacteria in TB patients is an important part of TB control project. Studies have shown that sputum culture which is still not negative at the end of 2 months is a predictor of treatment failure and recurrence [3]. There is also evidence that sputum culture conversion rate (SCC) may be an early predictor of successful treatment for multidrug-resistant (MDR-TB) [4, 5]. The acid-fast bacilli (AFB) smear-test result after 2 months treatment (second AFB smear-test) is great significance for the prediction of treatment outcome and evaluation of treatment management [6, 7].

Vitamin D_3_, also known as cholecalciferol [8] whose main effect is to maintain the function of monocytes and macrophages that play an important role in the pathogenesis related to human innate immunity. Vitamin D works by binding to the nuclear receptors of the affected cells, So the level of vitamin D in the human body will have an impact on the immune status of mycobacterium tuberculosis [9]. At present, the treatment of active tuberculosis is mainly antibacterial treatment, that is, the need for long-term use of high-dose chemotherapy drugs. Patients do not only face risk of drugs toxicity, but also possible drug resistance and high economic cost [10]. Vitamin D has a unique effect on the immune system, which makes it necessary to become an immunomodulatory therapy based on antibacterial treatment to shorten the duration of disease transmission [11], and to prevent the occurrence of drug resistance by shortening the time of chemotherapy. However, the mechanisms involved in the effect of vitamin D on TB is still unclear. In this paper, the relationship between second AFB smear-positive and vitamin D in TB patients was analyzed and inferred by the Bayesian network which is more sensitive to causality [12].

Bayesian network is a probability graph model proposed by Judea Pearl in 1985, which is an uncertainty processing model that simulates causality in the human reasoning process, and its topological structure is a directed acyclic graph. Each node in the directed acyclic graph represents a random variable, and the directed edge represents the conditional dependence between random variables [13]. Bayesian network model can clearly and intuitively express the interdependence between variables, which is more convenient for us to identify the probability causal dependence and conditional probability independence. Compared with the logistic regression model controlling confounding, Bayesian network can regulate the network through a priori experience, so as to give the objective conditional establishment probability, which makes the judgment more reasonable and accurate [14]. In this study, Bayesian network was used to explore the relationship between vitamin D level and the second AFB smear-positive among TB patients.

## 2 Materials and methods

### 2.1 Study design and ethics

**Fig 1:**
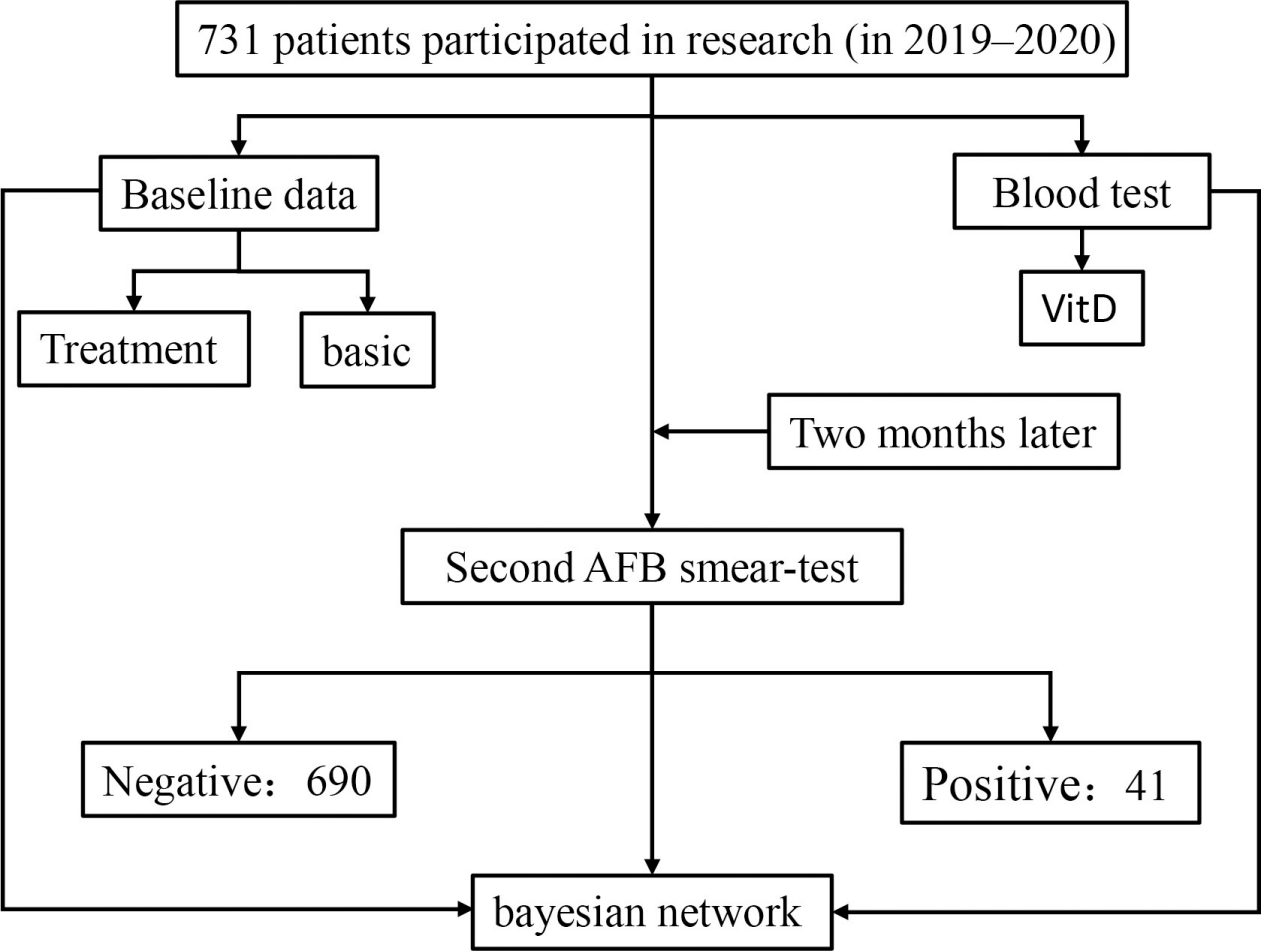
Flow chart of implementation process.

An epidemiological investigation by using a cross-sectional study design (Fig 1). The study plan was approved by the Ethics Committee of Xinjiang Medical University (No. K201910-07), and all the participants provided their voluntarily written informed consent before the investigation (Minors obtained the informed consent from their guardians).

### 2.2 Participants

The subjects of this study had been registered with Directly Observed Treatment of Short Course Strategy (DOTS) in Urumqi Center for Disease Control and Prevention (CDC) among December 2019~December 2020. All the patients were diagnosed, registered and treated by the Urumqi TB prevention and treatment institution. Inclusion criteria: Patients who were on standard TB treatment plan for two months and have completed with informed consent. Exclusion criteria: The main medical records were incomplete. A total of 731 patients have participated in this study.

### 2.3 Basic information collection

The inclusion and exclusion criteria were strictly observed during the data collection process. The basic medical record information of TB patients is derived from the TB special report information system. It includes basic demographic data such as gender, age, nationality, floating population and source of patients; Treatment related information of patients, such as treatment classification, diagnosis classification, baseline AFB smear-test results, patients with cavity, treatment management. The collected data were consistent with the patient’s medical records and registration books. After 2 months of treatment, second AFB smear-test was performed and medical records were recorded.

### 2.4 Vitamin D data collection

After obtaining informed consent, 5ml peripheral venous blood of patients was extracted by using disposable vacuum anticoagulant blood collection vessel when persons were diagnosed with TB. The serum plasma was separated, and the content of 25 hydroxy vitamin D which the active form of vitamin D that in patients’ serum was detected by Enzyme-linked immunosorbent assay (ELISA). 25-HVD3(25-Hydroxy Vitamin D3) ELISA Kit that Catalog No: E-EL-0015c; Intra-assay Precision (Coefficient of variation, CV):3.27%; Inter-assay Precision (CV):4.75%) was used in this study. In the detection of serum vitamin D level in TB patients, whose blood samples with severe hemolysis were excluded. In order to ensure the consistency of inspection results, the detection kit provided by the same company was used and the operation steps were strictly in accordance with the instructions.Serum-25-hydroxyvitamin-D of 0–29 ng/ml were considered as deficient, and ≥30 ng/ml as sufficient [15, 16].

### 2.5 Statistical analysis

Excel was used to capture the data, SPSS version 25.0 (IBM USA) was used for statistical analysis. chi-square test was used for comparison and correlation analysis; non-conditional logistic regression was used for multivariate analysis. Using the backward stepwise regression method, the test level of the included model was 0.05, and the exclusion standard was 0.10. The "forestplot" package in R 4.0.3 was used to draw forest map. tetrad 6.9.0 (NIH Big Data to Knowledge (BD2K) Consortium, USA) was used to construct the Bayesian network model and carry out the analysis.

## 3 Results

### 3.1 Second AFB smear-positive status among different TB patients

According to the results of the data analysis (Table 1), 41 (5.6%) of the 731 TB patients were second AFB smear-positive. Statistically significant differences were found in the detection rate of second AFB smear-positive among patients who differed in terms of their patient source, treatment classification, baseline sputum smear, combined cavity and treatment management (*P* < 0.05).

**Table 1:** Comparison of second AFB smear-positive status among different TB patients.

	Groups	Number	second AFB smear-test		χ^2^	*p*
			Positive	Incidence (%)		
gender	men	433	29	6.7	2.378	0.123
	women	298	12	4.0		
Age groups	≤30	213	9	4.2	1.563	0.458
	31~59	304	17	5.6		
	≥60	214	15	7.0		
Nation	Han	511	28	5.5	0.054	0.817
	Minority	220	13	5.9		
District	Suburbs	230	10	4.3	1.008	0.315
	Centre	501	31	6.2		
Address	Floating population	186	15	8.1	2.842	0.092
	Local population	545	26	4.8		
Patient source	track	390	17	4.4	6.290	0.043
	Referral	263	22	8.4		
	Other	78	2	2.6		
Treatment classification	Initial treatment	685	33	4.8	10.607	0.010
	Retreatment	46	8	17.4		
Diagnostic classification	Secondary	669	41	6.1	2.951	0.086
	Non secondary	62	0	0		
Baseline AFB smear-test	Negative	475	3	0.6	63.466	<0.001
	Positive	256	38	14.8		
Cavity	No	590	19	3.2	32.959	<0.001
	Yes	141	22	15.6		
Treatment management	Intensive supervision	426	2	0.5	60.050	<0.001
	Full supervision	224	34	15.2		
	Full management	81	5	6.2		

Patient source: TB patients are classified according to different sources; Track: Under the guidance of CDC, the primary medical institutions should follow up the TB patients who have not visited TB designated institutions or who have close contacts with suspicious symptoms, so that they can go to TB designated medical institutions for treatment; Referral: Refers to the suspected or confirmed TB patients at all levels of medical and health institutions transferred to TB designated medical institutions; Other: Including physical examination and who was close contact inspection. Treatment management: The management of TB patients in China is mainly based on non-hospitalization, including full supervision, intensive supervision, full management and self-medication; Intensive supervision: In the intensive period of treatment, patients were treated with direct supervision chemotherapy every time, and the full management was adopted in the continuous period; Full supervision: In the whole process of tuberculosis treatment, each time the patient takes anti-tuberculous drugs, they are under the direct supervision of medical staff or trained volunteer supervisors; Full management: In the whole process of patient treatment, urge patients to complete the whole course of treatment through various ways and channels.

### 3.2 Logistic regression analysis of second AFB smear-positive

**Fig 2:**
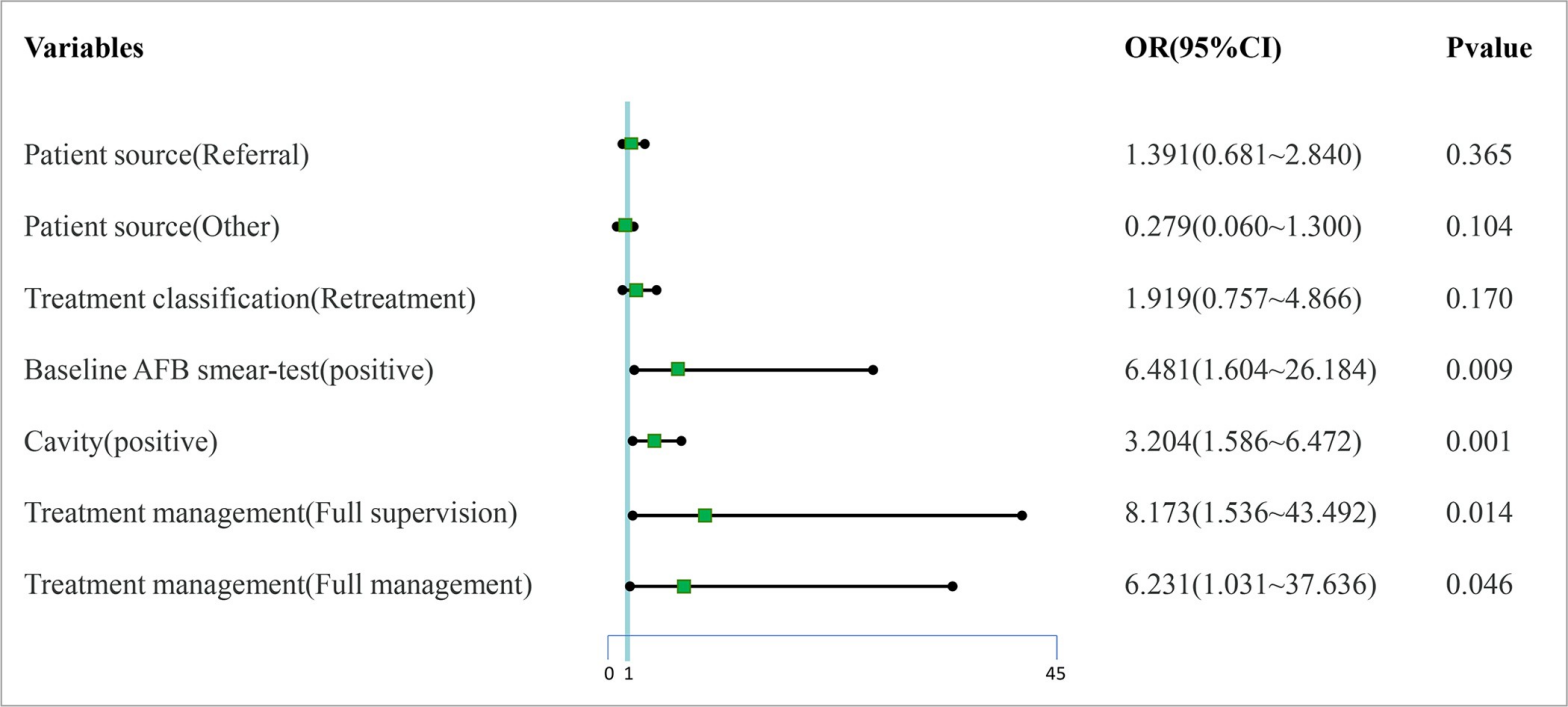
The result of logistic regression.

The dependent variable was the result of the second AFB smear-test, and the independent variable was the significant individual factor in the univariate analysis. The independent variables mainly included patient source, treatment classification, baseline sputum smear, cavity and treatment management (Table 2). The results showed that Baseline sputum smear, Cavity, Treatment management could affect the rate of second AFB smear-positive (*P*<0.05). Among them, baseline AFB smear-positive, combined cavity, Full supervision mode and Full management mode were risk factors for second AFB smear-positive (Fig 2).

**Table 2:** Assignment table of dependent variable and independent variables.

Variable type	Variables	Assignment
Dependent variable	Second AFB smear-test	Negative = 0 Positive = 1
Independent variable	Patient source	Track = (0,0) Referral = (1,0) Other = (0,1)
	Treatment classification	Initial treatment = 0 retreatment = 1
	Baseline AFB smear-test	Negative = 0 Positive = 1
	Cavity	No = 0 Yes = 1
	Treatment management	Intensive supervision = (0,0)
		Full supervision = (1,0)
		Full management = (0,1)

### 3.3 Second AFB smear-positive of TB patients with different vitamin D levels

There were 316(43.2%) with vitamin D sufficient and 415 (56.8%) with vitamin D deficiency among 731 patients. There was significant statistically difference in the rate of second AFB smear-positive among TB patients with different levels of vitamin D (*P* < 0.05). The rate of second AFB smear-positive in vitamin D sufficient group (7.6%) was higher than that in vitamin D deficiency group (4.5%) (Table 3).

**Table 3:** Second AFB smear-test results of TB patients with different vitamin D levels.

Vitamin D levels	Second AFB smear-test		χ2	P
	Positive	Incidence (%)		
Sufficient	24	7.6	4.148	0.042
Deficiency	17	4.5		

### 3.4 Construction of a Bayesian network model for second AFB smear-positive

**Fig 3:**
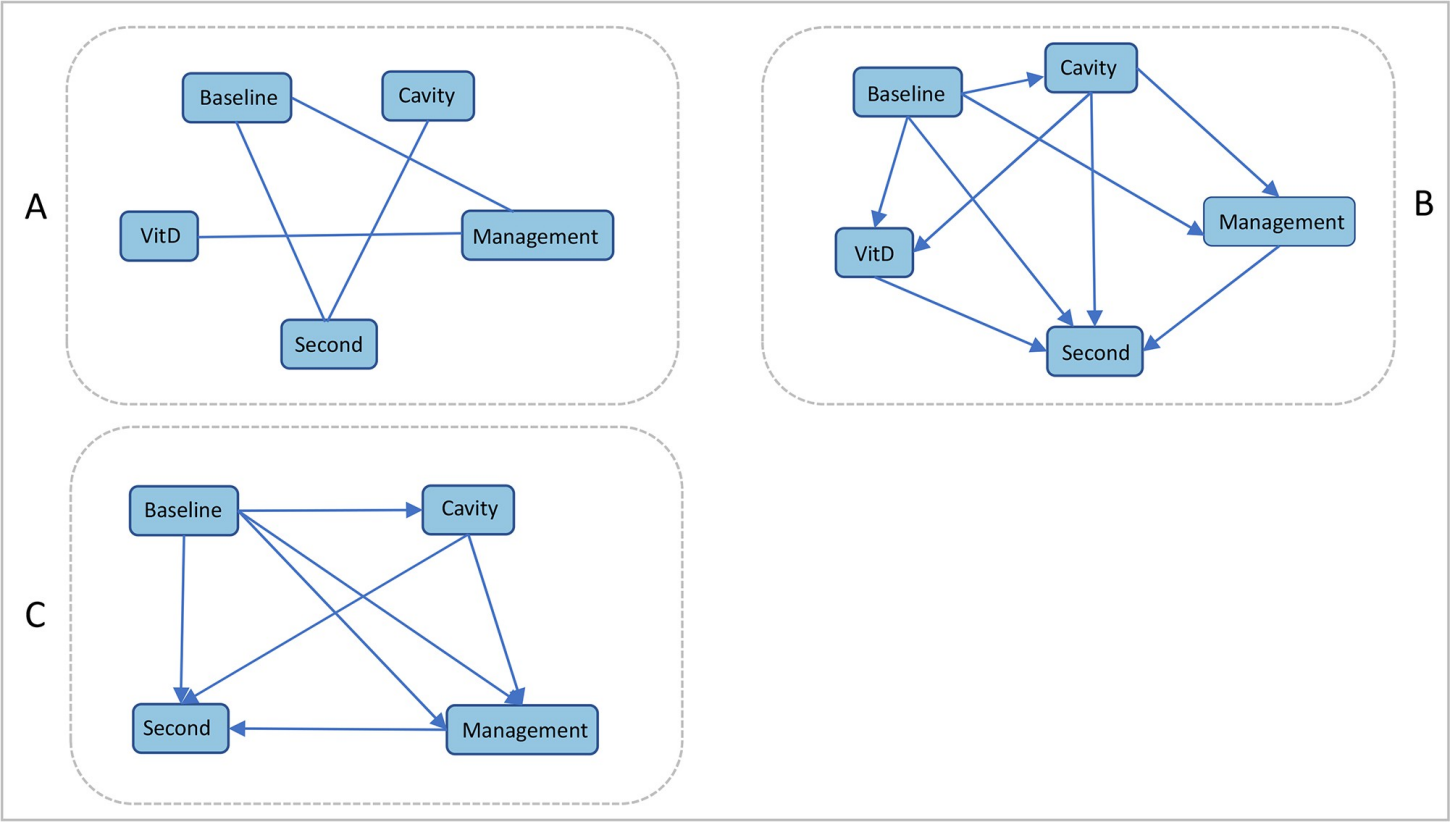
Bayesian network model for second AFB smear-positive in TB patients. **A**: Initial network structure. **B**: Bayesian network structure after adding constraints according to prior knowledge. **C**: Trimmed Bayesian network structure.

In this study, tetrad6.9.0 software was used to construct Bayesian network. The influencing factors (*P* < 0.05) of second AFB smear-positive were as follows: baseline sputum smear, cavity, treatment management mode and vitamin D level of TB patients were used as network nodes to construct Bayesian network model. The definition and assignment of Bayesian network nodes were shown in Table 4. There were three steps to build Bayesian network structure (Fig 3). The data set of model construction was discrete data, PC variants algorithm was used in the whole calculation process, the test method was chi-square test, and the conditional independent test level was alpha < 0.05.

**Table 4:** The definition and assignment table of Bayesian network nodes.

Variables	node	Assignment
Baseline AFB smear-test	Baseline	Negative = 0 Positive = 1
Cavitary	Cavity	No = 0 Yes = 1
Treatment management	Management	Intensive supervision = 0
		Full supervision = 1
		Full management = 2
Vitamin D levels	VitD	Sufficient = 0 Deficiency = 1
Second AFB smear-test	Second	Negative = 0 Positive = 1

**Fig 4:**
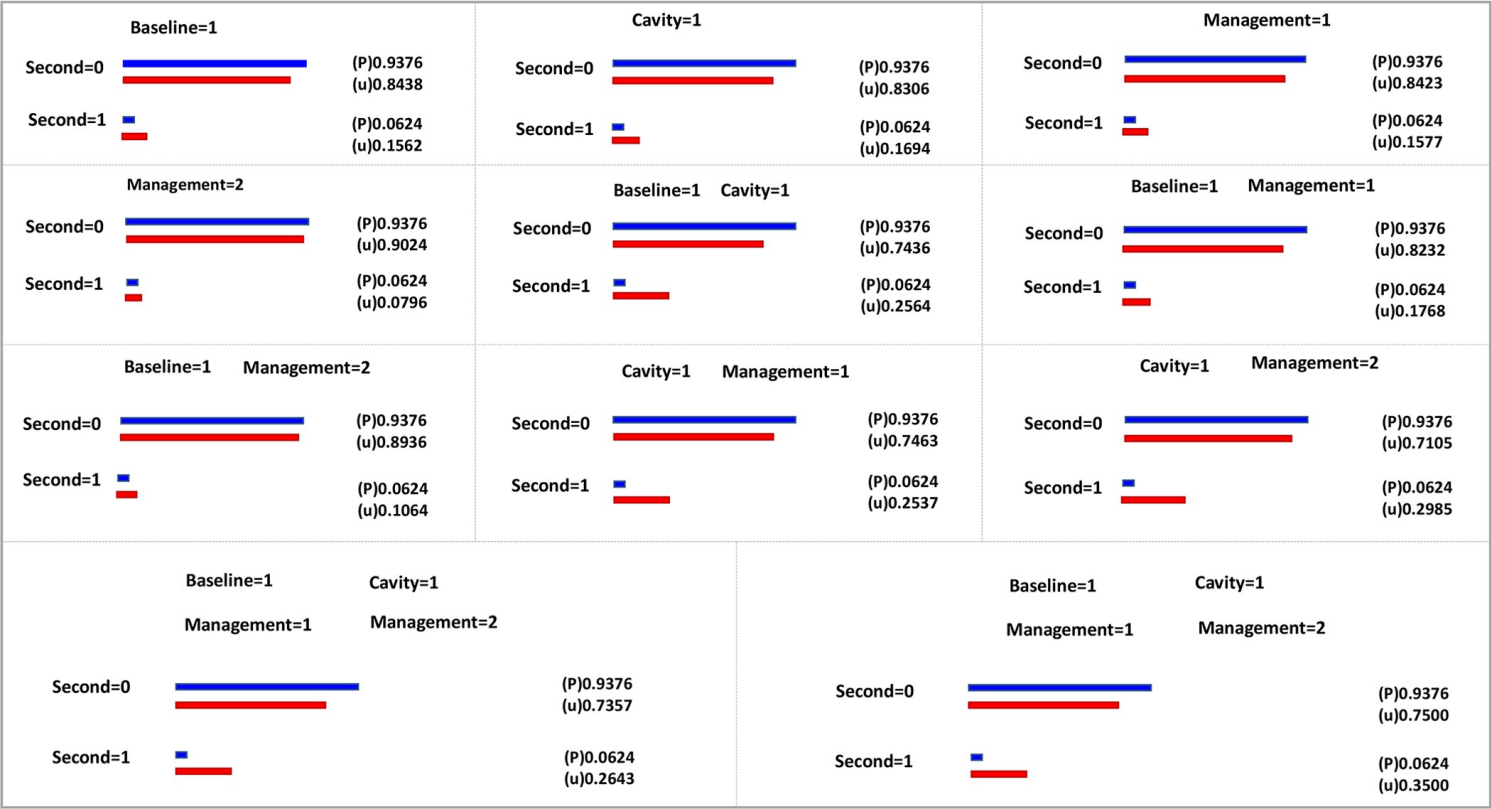
Conditional probability table of second AFB smear-positive.

As shown in Fig 3, Graph A was to explore the initial model from the original data. At this time, the graph had no arrow pointing, and the connection only indicated the correlation between the two variables, so it was necessary to further add constraints to the model; Graph B was the graph that constraints were imposed on the model according to the chronological order of independent variables and calculated the edge probability of each variable (Table 5), where the probability of “Baseline to VitD”, “Cavity to VitD”, “VitD to Second” three edges were all less than 0.5, and the vitamin D level of TB patients was less likely to affect the second AFB smear-positive (Ensemnle = 0.0709). It was not considered to be the interpretable cause of second AFB smear-positive; After eliminating the edges with edge probability less than 0.5, reconstructed the model (Graph C) and calculated the conditional probability of positive in second AFB smear-positive (Fig 4). Baseline AFB smear-positive, combined cavity and different treatment management mode all had a direct impact on the second sputum smear, which was consistent with the logistic regression results, and the logistic regression results were supplemented in more detail through the conditional probability table (Fig 4).

**Table 5:** Edges and edge type probabilities of Bayesian network node.

Node 1	Interaction	Node 2	Ensemnle	No dege
Baseline	**→**	Cavity	1.0000	0.0000
Baseline	**→**	Management	1.0000	0.0000
Baseline	**→**	VitD	0.0340	0.9660
Baseline	**→**	Second	0.8791	0.1209
Cavity	**→**	Management	0.7852	0.2148
Cavity	**→**	VitD	0.1568	0.8432
Cavity	**→**	Second	0.8252	0.1748
Management	**→**	Second	0.8302	0.1698
VitD	**→**	Second	0.0709	0.9291

## 4 Discussion

Baseline AFB smear-positive patients were more likely to continue to be positive in the second AFB smear-test. The baseline AFB smear-positive patients who carry more Mycobacterium tuberculosis are due to the long delay of treatment time. A retrospective study of 330 patients with drug resistance from Yangon of Burma showed that delayed initiation of treatment was associated with poor treatment outcome. Median treatment-delay times were longer among TB patients with poor outcomes (144 days) than those with successful outcomes (102 days). Patients with long treatment delays were significantly different from those with short delays, in terms of having high sputum smear grade, resistance to more than two main drugs (isoniazid and rifampicin), and long culture conversion time [17]. On the other hand, patients with different constitution and related gene expression, such as the presence of susceptibility genes in patients is related to persistent positive sputum smear. A cohort study of Vitamin D receptor (VDR) gene polymorphisms showed that the VDR gene polymorphisms were associated with rate of sputum culture conversion in MDR TB patients [18]. Research shows that patients with the *TT TaqI* genotype had significantly longer time to sputum culture conversion compared with *Tt* genotype (median 46 days for *TT* genotype vs. 16 days for *Tt* genotype) [19]. And other report showed that a significantly lower proportion of patients with *ApaI* aa genotype had converted cultures by 2 months of TB treatment compared to patients with *ApaI* Aa genotype (26% vs. 51%, *P* = 0.03) [20]. In addition, it may be that the baseline AFB smear-positive patients are insensitivity to anti-tuberculosis drugs [21]. And two months of treatment can not completely turn most baseline sputum smear positive patients into negative.

TB patients with cavities are often more severe and have higher mycobacterial load, initial bacterial load is associated with sputum smear delayed conversion to negative at the end of TB treatment [22]. A review published of *LANCET INFECTIOUS DISEASES* in 2020 pointed out: Phagocytes and granulocytes penetrate poorly went into these necrotic areas(cavity), thus creating an immune-sheltered zone of bacterial growth. High oxygen levels within the cavity also provided a rich environment for high rates of bacterial replication leading to a large bacillary burden at the inner edge of the cavity. During cavity formation, both the basement membrane and alveolar architecture were permanently destroyed. Even after successful TB treatment, TB cavities can persist, leading to lifelong pulmonary deficits and recurrent opportunistic infections. All the above reasons lead to the characteristics of cavitary tuberculosis, which is difficult to cure completely and easy to relapse [23]. Many studies have shown that the presence of cavities in chest X-ray films is a risk factor for tuberculosis recurrence. Patients with cavities are more likely to have adverse treatment outcomes than patients without cavities. It had negative implications not only for the patient-associated with poor treatment outcomes, including delayed sputum culture conversion, relapse after treatment, and development of drug resistance, but was also a public health threat, which since cavitation greatly increased the risk of person-to-person transmission [24, 25]. Therefore, it is necessary to improve the early detection rate of TB patients to avoid the deterioration of the disease and the formation of cavities [26]. At the same time, it should be ensured that the cavity disappears at the end of treatment, and regular follow-up should be conducted for patients who still have cavity at the end of treatment.

The different treatment management patterns also had different effects on second AFB smear-test results and treatment outcomes of TB patients [27]. Most countries gave some forms of TB patients support including various treatment supervision options and treatment adherence interventions such as health education, psycho-emotional and socio-economic support [28]. The full supervision mode is suitable for AFB smear-positive patients, new AFB smear-negative patients with miliary cavity and other serious complications. The disease situation of these patients is relatively serious, so the second AFB smear-positive rate is higher. However, the full management refers to the whole process of tuberculosis treatment for patients who have not reached the full supervision or intensive supervision. Through all the comprehensive management methods such as strengthening the propaganda and education of patients, regular outpatient medication, family visits, return visit medication situation, delayed recovery to ensure the regular medication of patients. For the TB patients, regular medication is an important condition to improve the disease and prognosis [29]. TB patients should complete the whole treatment process according to the medical advice and should not interrupt or shorten the medication time and need to take medicine for a long time. Only regular medication on time can ensure the effective dose of the drug concentration in the body and make the drug continue to play its role. However, some of the patients who received the full management stopped treatment by themselves after their symptoms improved significantly, or some of them stopped taking drugs by themselves because of adverse drug reactions and poor economic ability, resulting in a higher rate of the second AFB smear-positive [30, 31]. Patients who received two or more anti TB courses were significantly associated with multidrug-resistant tuberculosis compared with patients receiving an anti TB course. In some instances, individualizing treatment regimens may be necessary [32].

The results of this study do not believe that vitamin D deficiency in TB patients is the cause of AFB smear-positive after 2 months treatment, which is consistent with the results of a meta-analysis from China. The meta-analysis considered that vitamin D supplementation did not shorten the time to sputum culture and smear conversion and did not lead to an increase in the proportion of participants with negative sputum culture [33]. However, there is still no unified statement on the role of vitamin D level of TB patients in TB treatment, and there are many reasons for this situation. The first is the effect of chemotherapy drugs on their own metabolism during treatment. Such as Rifampicin (RFP) limits the formation of active form of vitamin D in anti-tuberculosis treatment, and Isoniazid (INH) leads to the reduction of corresponding vitamin D metabolites, so that the vitamin D level of patients generally decreases during treatment [34, 35]. In addition, the relationship between vitamin D and the outcome of TB treatment was different in different study populations. A meta-analysis showed that the level of vitamin D had no significant effect on the time of sputum culture transformation in general TB patients, but it played an accelerating role in the sputum culture transformation of MDR-TB patients [36]. Finally, the vitamin D level of TB patients may be affected by genetic factors, such as black Americans had low levels of vitamin D compared with whites [37]. And different combinations of vitamin D levels and genotypes of vitamin D receptor gene polymorphisms have different sputum culture conversion rates in TB patients [38].

## 5 Conclusion

The risk factors of second AFB smear-positive were baseline AFB smear-positive, combined cavity, full supervision and full management. Inference results based on Bayesian network, the vitamin D level in TB patients was not considered as one of the reasons for the AFB smear-positive. Baseline AFB smear-positive as well as cavity caused by delayed treatment should be avoided and the medication compliance education of patients in the full management should be strengthen in the process of TB prevention and control.

## 6 Limitation

First, the research object of this study is only from tuberculosis patients in Urumqi, so it is not clear whether the research results are applicable to other regions and countries. Secondly, AFB smear-test can be classified data. Due to the limitation of conditions, it is regarded as secondary classification data in this study. In the follow-up research, we should try to ensure the originality and comprehensiveness of AFB-test result. Third, this study adopts cross-sectional design, which can only use statistical techniques to infer the preliminary etiological clues from the probability rather than identify a clear causal relationship. It is suggested that the follow-up study can design community intervention trials, and further clarify the role of vitamin D in the treatment of tuberculosis.

## Supporting information

S1 Data (XLS)

## References

[1] Organization WH. Global tuberculosis report 2019. 2019;https://apps.who.int/iris/handle/

[2] World Health Organization Official accounts. Tuberculosis prevention and control in China.2020.https://baijiahao.baidu.com/s?id=1662089567052587845&wfr=spider&for=pc

[3] Van LH, Phu PT, Vinh DN, Son VT, Hanh NT, Nhat LTH, et al. Risk factors for poortreatment outcomes of 2266 multidrug-resistant tuberculosis cases in Ho Chi Minh City: a retrospective study. BMC Infect Dis. 2020;20(1):10. doi: 10.1186/s12879-019-4719-3 32087682 PMC7036193

[4] Basit A, Ahmad N, Khan AH, Javaid A, Sulaiman SAS, Afridi AK, et al. Predictors of Two Months Culture Conversion in Multidrug-Resistant Tuberculosis: Findings from a Retrospective Cohort Study. Plos One. 2014;9(4):6. doi: 10.1371/journal.pone.0093206 24705411 PMC3976287

[5] Rodriguez M, Monedero I, Caminero JA, Encarnacion M, Dominguez Y, Acosta I, et al. Successful management of multidrug-resistant tuberculosis under programme conditions in the Dominican Republic. Int J Tuberc Lung Dis. 2013;17(4):520–5. doi: 10.5588/ijtld.12.0481 23485386

[6] Lv LS, Li TC, Xu K, Shi PY, He BY, Kong WM, et al. Sputum bacteriology conversion and treatment outcome of patients with multidrug-resistant tuberculosis. Infection and Drug Resistance. 2018;11:147–54. doi: 10.2147/IDR.S153499 WOS:000423547500001. 29416359 PMC5790105

[7] Soeroto AY, Pratiwi C, Santoso P, Lestari BW. Factors affecting outcome of longer regimen multidrug-resistant tuberculosis treatment in West Java Indonesia: A retrospective cohort study. Plos One. 2021;16(2). doi: 10.1371/journal.pone.0246284 WOS:000617379900059. 33556094 PMC7870080

[8] Haussler MR, Whitfield GK, Haussler CA, Hsieh JC, Thompson PD, Selznick SH, et al. The nuclear vitamin D receptor: biological and molecular regulatory properties revealed. J Bone Miner Res. 1998;13(3):325–49. Epub 1998/04/03. doi: 10.1359/jbmr.1998.13.3.325 9525333

[9] Nnoaham KE, Clarke A. Low serum vitamin D levels and tuberculosis: a systematic review and meta-analysis. Int J Epidemiol. 2008;37(1):113–9. doi: 10.1093/ije/dym247 18245055

[10] Tweed CD, Crook NM, Amukoye EI, Dawson R, Diacon AH, Hanekom M, et al. Toxicity associated with tuberculosis chemotherapy in the REMoxTB study. BMC Infect Dis. 2018;18:11. doi: 10.1186/s12879-017-2932-5 29996783 PMC6042413

[11] Sassi F, Tamone C, D’Amelio P. Vitamin D: Nutrient, Hormone, and Immunomodulator. Nutrients. 2018;10(11):14. doi: 10.3390/nu10111656 30400332 PMC6266123

[12] Ma H, Xu CF, Shen Z, Yu CH, Li YM. Application of Machine Learning Techniques for Clinical Predictive Modeling: A Cross-Sectional Study on Nonalcoholic Fatty Liver Disease in China. Biomed Res Int. 2018;2018:9. doi: 10.1155/2018/4304376 30402478 PMC6192080

[13] Li X, Jiang T, Sun X, Yong X, Ma X, Liu J. The relationship between occupational stress, musculoskeletal disorders and the mental health of coal miners: The interaction between BDNF gene, TPH2 gene polymorphism and the environment. J Psychiatr Res. 2021;135:76–85. Epub 2021/01/16. doi: 10.1016/j.jpsychires.2020.12.061 33450468

[14] Pearl Judea, Mackenzie Dana. The Book of Why. 1st ed. New York: Penguin;2019.

[15] Krist AH, Davidson KW, Mangione CM, Cabana M, Caughey AB, Davis EM, et al. Screening for Vitamin D Deficiency in Adults: US Preventive Services Task Force Recommendation Statement. Jama. 2021;325(14):1436–42. Epub 2021/04/14. doi: 10.1001/jama.2021.3069 . 33847711

[16] Liao X, Zhang Z, Zhang H, Zhu H, Zhou J, Huang Q, et al. Application guideline for vitamin D and bone health in adult Chinese (2014 standard edition). Chin J Osteoporos. 2014;20(9):1011–30.

[17] Htun YM, Khaing TMM, Aung NM, Yin Y, Myint Z, Aung ST, et al. Delay in treatment initiation and treatment outcomes among adult patients with multidrug-resistant tuberculosis at Yangon Regional Tuberculosis Centre, Myanmar: A retrospective study. Plos One. 2018;13(12):20. doi: 10.1371/journal.pone.0209932 30596734 PMC6312206

[18] Magee MJ, Sun YV, Brust JCM, Shah NS, Ning YM, Allana S, et al. Polymorphisms in the vitamin D receptor gene are associated with reduced rate of sputum culture conversion in multidrugresistant tuberculosis patients in South Africa. Plos One. 2017;12(7):11. doi: 10.1371/journal.pone.0180916 28700743 PMC5507304

[19] Roth DE, Soto G, Arenas F, Bautista CT, Ortiz J, Rodriguez R, et al. Association between vitamin D receptor gene polymorphisms and response to treatment of pulmonary tuberculosis. J Infect Dis. 2004;190(5):920–7. Epub 2004/08/06. doi: 10.1086/423212 15295697

[20] Babb C, van der Merwe L, Beyers N, Pheiffer C, Walzl G, Duncan K, et al. Vitamin D receptor gene polymorphisms and sputum conversion time in pulmonary tuberculosis patients. Tuberculosis. 2007;87(4):295–302. doi: 10.1016/j.tube.2007.03.001 17449323

[21] Na HH, Ryu JM, Kim KC. Knockout of FosB gene changes drug sensitivity and invasion activity via the regulation of Bcl-2, E-cadherin, fi-catenin, and vimentin expression. Biochem Biophys Res Commun. 2021;567:131–7. doi: 10.1016/j.bbrc.2021.06.031 34153682

[22] Grozdanovic Z, Almanza LCB, Goyal S, Hussain A, Klassert TE, Driesch D, et al. A Novel Reading Scheme for Assessing the Extent of Radiographic Abnormalities and Its Association with Disease Severity in Sputum Smear-Positive Tuberculosis: An Observational Study in Hyderabad/India. Plos One. 2015;10(9):16. doi: 10.1371/journal.pone.0138070 26381644 PMC4575099

[23] Urbanowski ME, Ordonez AA, Ruiz-Bedoya CA, Jain SK, Bishai WR. Cavitary tuberculosis: the gateway of disease transmission. Lancet Infect Dis. 2020;20(6):e117–e28. Epub 2020/06/03. doi: 10.1016/S1473-3099(20)30148-1 32482293 PMC7357333

[24] Palaci M, Dietze R, Hadad DJ, Ribeiro FKC, Peres RL, Vinhas SA, et al. Cavitary disease and quantitative sputum bacillary load in cases of pulmonary tuberculosis. J Clin Microbiol. 2007;45(12):4064–6. doi: 10.1128/JCM.01780-07 17928422 PMC2168542

[25] Benator D, Bhattacharya M, Bozeman L, Burman W, Cantazaro A, Chaisson R, et al. Rifapentine and isoniazid once a week versus rifampicin and isoniazid twice a week for treatment of drug-susceptible pulmonary tuberculosis in HIV-negative patients: a randomised clinical trial. Lancet. 2002;360(9332):528–34. Epub 2002/09/21. doi: 10.1016/s0140-6736(02)09742-8 12241657

[26] Kempker RR, Rabin AS, Nikolaishvili K, Kalandadze I, Gogishvili S, Blumberg HM, et al. Additional Drug Resistance in Mycobacterium tuberculosis Isolates From Resected Cavities Among Patients With Multidrug-Resistant or Extensively Drug-Resistant Pulmonary Tuberculosis. Clinical Infectious Diseases. 2012;54(6):E51–E4. doi: 10.1093/cid/cir904 22198790 PMC3284212

[27] Fang XH, Shen HH, Hu WQ, Xu QQ, Jun L, Zhang ZP, et al. Prevalence of and Factors Influencing Anti-Tuberculosis Treatment Non-Adherence Among Patients with Pulmonary Tuberculosis: A Cross-Sectional Study in Anhui Province, Eastern China. Med Sci Monitor. 2019;25:1928–35. doi: 10.12659/MSM.913510 30869079 PMC6429981

[28] Jansen-Aaldring N, van de Berg S, van den Hof S. Patient support during treatment for active tuberculosis and for latent tuberculosis infection: Policies and practices in European low-incidence countries. J Adv Nurs. 2018;74(12):2755–65. doi: 10.1111/jan.13784 29964334

[29] Arozal W, Diliana, Wikanendra GB, Purwantyastuti, Rusli A. Clinical characteristics of recurrent tuberculosis patients from a Jakarta hospital-based survey. J Pak Med Assoc. 2021;71(2):S58–S61. 33785943

[30] Mukasa J, Kayongo E, Kawooya I, Lukoye D, Etwom A, Mugabe F, et al. Adherence to the MDR-TB intensive phase treatment protocol amongst individuals followed up at central and peripheral health care facilities in Uganda—a descriptive study. Afr Health Sci. 2020;20(2):625–32. doi: 10.4314/ahs.v20i2.10 33163023 PMC7609083

[31] Zhang JJ, Yang YY, Qiao X, Wang LW, Bai JY, Yangchen T, et al. Factors In fluencing Medication Nonadherence to Pulmonary Tuberculosis Treatment in Tibet, China: A Qualitative Study from the Patient Perspective. Patient Prefer Adherence. 2020;14:1149–58. doi: 10.2147/PPA.S252448 32764888 PMC7360411

[32] Shah M, Reed C. Complications of tuberculosis. Curr Opin Infect Dis. 2014;27(5):403–10. doi: 10.1097/QCO.0000000000000090 25028786

[33] Zhang J, Chen C, Yang J. Effectiveness of vitamin D supplementation on the outcome of pulmonary tuberculosis treatment in adults: a meta-analysis of randomized controlled trials. Chin Med J. 2019;132(24):2950–9. doi: 10.1097/CM9.0000000000000554 31833904 PMC6964947

[34] Wang Z, Lin YS, Zheng XE, Senn T, Hashizume T, Scian M, et al. An inducible cytochrome P450 3A4-dependent vitamin D catabolic pathway. Mol Pharmacol. 2012;81(4):498–509. Epub 2011/12/30. doi: 10.1124/mol.111.076356 22205755 PMC3310418

[35] Ralph AP, Lucas RM, Norval M. Vitamin D and solar ultraviolet radiation in the risk and treatment of tuberculosis. Lancet Infect Dis. 2013;13(1):77–88. Epub 2012/12/22. doi: 10.1016/S1473-3099(12)70275-X 23257233

[36] Jolliffe DA, Ganmaa D, Wejse C, Raqib R, Haq MA, Salahuddin N, et al. Adjunctive vitamin D in tuberculosis treatment: meta-analysis of individual participant data. Eur Resp J. 2019;53(3):13. doi: 10.1183/13993003.02003-2018 30728208

[37] Powe CE, Evans MK, Wenger J, Zonderman AB, Berg AH, Nalls M, et al. Vitamin D-Binding Protein and Vitamin D Status of Black Americans and White Americans. New England Journal of Medicine. 2013;369(21):1991–2000. doi: 10.1056/NEJMoa1306357 WOS:000327163000007. 24256378 PMC4030388

[38] Martineau AR, Timms PM, Bothamley GH, Hanifa Y, Islam K, Claxton AP, et al. High-dose vitamin D(3) during intensive-phase antimicrobial treatment of pulmonary tuberculosis: a double-blind randomised controlled trial. Lancet. 2011;377(9761):242–50. Epub 2011/01/11. doi: 10.1016/S0140-6736(10)61889-2 ; PubMed Central PMCID: PMC4176755. 21215445 PMC4176755

